# Observations on the Sickness Which Prevailed at Jessore, (Bengal), in the Months of Aug. and Sept. 1817

**Published:** 1818-10-01

**Authors:** Robert Tytler, William Evans

**Affiliations:** Assistant Surgeon, Civil Station, Zilla, Jessore; Surgeon, Royal Navy


### *Observations on the Sickness which prevailed at Jessore, (Bengal), in the Months of Aug. and Sept.* 1817.

By Robert Tytler, M. D. Assistant Surgeon, Civil Station, Zilla, Jessore.

[Communicated by William Evans, Esq. Surgeon, Royal Navy.]

THE situation allotted for the station, comprehending a few brick-built, scattered houses, chiefly lower roomed, or consisting of one story, but well elevated and flued, and inhabited by the Europeans, is a flat spot of ground, intersected with ditches, and containing much jungle, along with indigo and paddy fields, reaching close upon the premises surrounding the habitations. On the north side is an inconsiderable branch of the Ganges, having little communication with the parent river, and which, except in the rainy season, is almost stagnant. On the *northern* side of this, opposite to the town, a broad space of low and sunk ground is left uncovered, and, during the wet months, converted into a stagnant offensive marsh. Along the *southern* bank the Bazar runs in a continued line for a space of more than two miles, consisting of narrow and damp streets, lined with huts, crowded upon each other, and containing, for sale, various putrescent articles of food, emitting disagreeable and noxious effluvia. The walls of these huts are constructed of bamboos and straw. They are perforated by very narrow windows, have but one small door, and consequently free ventilation of atmospheric air never takes place within their dismal interiors.

From the end of January, much rain fell, which is rather an unusual occurrence in Bengal; in consequence of which the lower grounds were, in several places, inundated, and by the solar heat a considerable extrication of miasmata succeeded, and some mortality prevailed, but not to any great extent.

On the 19th of August, when the prevalence of a general disease was expected, a case of severe illness was reported to me by a native doctor. I found a middle-aged man in a state of great prostration of strength, surrounded by his friends, who were pouring water into his mouth. The preceding day he was in perfect health. In the night, he had been seized with violent abdominal pains, repeated vomiting, frequent discharges from the bowels, and incessant thirst. The symptoms in the present case were precisely the same with those since ascertained to mark this peculiar disorder. The face exhibited a livid paleness (indicative of great corporeal distress in a native); the eyes were hollow; the eyelids half closed; the forehead bedewed with cold perspiration; the extremities and surface of the body frigid; no vestige of pulse at the wrists or temples.* He died the next day; and it was now ascertained that seventeen had perished in the same manner during the two or three preceding days.

The rapid progress of the disorder now produced universal alarm among the natives; and I find, by my notes, that on the 21st of August fifteen men died in the Bazar, all affected with the same symptoms; viz. violent gripings, vomiting, and purging, which, if not relieved soon, generally terminated fatally in a few hours.

As the usual modes of treatment employed in cholera morbus afforded little prospect of benefit, I ventured to have recourse to calomel and opium, and the success of the practice exceeded all expectation, especially when exhibited early, and before the constitution became so enervated, that no hope existed of recalling the flitting spark of life.

In order to obviate this nervous depression, I resolved to administer a volatile antispasmodic mixture, containing two ounces of æther, the same quantity of spirits of ammonia, and a quart of water. This mixture proved of the greatest benefit. These were the only remedies employed by myself, and under my own directions by the native assistants in the town of Cushbah, its vicinity, and the

---

* Dr. Tytler here, and throughout the paper, labours to trace the cause of this epidemic to damaged rice; but the reasonings are so bad, and the facts so dubious, that we entirely omit them. The causes of this epidemic will be more rationally accounted for in the succeeding paper, by Mr. Evans. *Editor.*

jail; and, notwithstanding the hurry and confusion consequent on so extended an epidemic, the number rescued from the jaws of death by these means, considerably exceeds two hundred, most of whom are now living at Jessore.

The method of treatment was this. Eight grains of calomel were invariably administered at the first visit, followed by the opium in doses of a grain, and the antispasmodic mixture, as the reduction of pulse, and frigid feel of the skin, indicated its exhibition, and the continuance of the calomel at intervals, in doses of four grains, till the virulence of the symptoms was subdued. In some cases, the calomel was taken to the amount of 30 grains, in the course of twenty-four hours; the opium to four grains in the same time; and the mixture, containing the ammonia and æther, eight ounces. But in general, upon swallowing the dose of calomel, the vomiting ceased, and the patients were relieved.

*Pathology.* Only one dissection could be procured—a convict in the jail. The mucous membrane of the stomach and bowels was inflamed.

---

The following observations on this Epidemic, as it appeared in Calcutta and the neighbourhood, were obligingly communicated by Mr. William Evans, who was an eye-witness.

" The year 1817 was uncommonly irregular. The rains set in about the middle of February, and continued till the night of the 17th of October, concluding with a heavy fall. The thermometer, during all this wet season, ranged from 85 to 98°. The whole country became inundated, was one vast swamp, and sent forth the most pestilential miasmata. The number of natives who fell victims to this disease at Calcutta was almost incalculable. Several hundred bodies were burnt every night at some of the Ghauts leading from Chitpore road to the Hoogly, and the ashes thrown into the river. There were full as many thrown into the water, for want of sufficient fuel to burn them. The floating corses at length became so numerous, and so entangled among the cables of the shipping, that the stench was unbearable, and the magistrates were forced to employ Mussulmen to clear the bodies away.

" The environs of Calcutta exhibited, towards the latter end of October, the most appalling scenes of distress.

Most of the palankeen bearers and coolies fled to the country in hopes of escaping the sickness; and by crowding into obscure villages, spread the epidemic in all directions. But few of the Europeans suffered, evidently owing to their more comfortable lodgings, and superior salubrity of food, clothing, &c.

"About the 20th of October the monsoon changed to the north-east, with a cold piercing wind; the ground still wet. The disease now quickly changed its type from cholera to dysentery, which became milder and milder to the end of November, when it ceased altogether. At this time the country was dry.

"During August and September, cases of fever were not unfrequent, and several Europeans were cut off in a few hours by the violence of the disease. At Calcutta the attack frequently came on instantaneously, with large frothy stools; vomiting of limpid fluid, sometimes mixed with bile; great prostration of strength; distortion of countenance, which changed from black to a deep lead colour. The eyes were sunk and their lustre lost; immediately on the accession of the disease, the pupil became dilated, and appeared as though the patient were dead. I have seen them fall down in the streets, faint, and often die forthwith!

"The opium and calomel pills, as recommended by Dr. Tytler, were used at Calcutta, and certainly with benefit. I found it better to give the tincture of opium with calomel in a jelly, and some warm aromatic cordial immediately afterwards. But the "*Drogue Mere*," of which I send you the recipe, was the most beneficial of all remedies, and had an immense sale latterly. The vender and preparer was an old Spanish Padre (Dr. Minguet), with whom I was intimate, and who kindly furnished me with a specimen of the plant herewith transmitted. The dose of the medicine was a small wine glass full occasionally.

"FORMULA.

"℞. Aloes - - - - - - - Oiij;

Myrrhæ - - - - - - Oij;

Gum. Olibani - - - Oj;

Croci - - - - - - - ℥iv;

Herbæ justiciæ paniculatæ Oj;

Aquæ vitæ - - - - OL.

Ft. infusio.

"WILLIAM EVANS.

"*London, July* 1818."

